# Meta-Network Analysis of Structural Correlation Networks Provides Insights Into Brain Network Development

**DOI:** 10.3389/fnhum.2019.00093

**Published:** 2019-03-26

**Authors:** Xiaohua Xu, Ping He, Pew-Thian Yap, Han Zhang, Jingxin Nie, Dinggang Shen

**Affiliations:** ^1^Department of Computer Science, Yangzhou University, Yangzhou, China; ^2^Department of Radiology and BRIC, University of North Carolina at Chapel Hill, Chapel Hill, NC, United States; ^3^School of Psychology, South China Normal University, Guangzhou, China; ^4^Department of Brain and Cognitive Engineering, Korea University, Seoul, South Korea

**Keywords:** brain network development, cortical thickness, meta-network analysis, low rank, temporal smoothness

## Abstract

Analysis of developmental brain networks is fundamentally important for basic developmental neuroscience. In this paper, we focus on the temporally-covarying connection patterns, called meta-networks, and develop a new mathematical model for meta-network decomposition. With the proposed model, we decompose the developmental structural correlation networks of cortical thickness into five meta-networks. Each meta-network exhibits a distinctive spatial connection pattern, and its covarying trajectory highlights the temporal contribution of the meta-network along development. Systematic analysis of the meta-networks and covarying trajectories provides insights into three important aspects of brain network development.

## Introduction

Over the past decade, the neuroscience community has reached the consensus that human brain development is a structurally and functionally non-linear process (Gogtay et al., 2004). The understanding of normal brain development is essential for understanding the neurodevelopmental disorders, such as autism spectrum disorder (Bray, 2017), schizophrenia (Fan et al., 2008; Franke et al., 2016), and attention deficit hyperactivity disorder (ADHD) (Kuntsi et al., 2005).

In recent years, studies on the development of brain networks have gained increasing attention. Rapidly evolving technologies, such as Magnetic Resonance Imaging (MRI), Diffusion Tensor Imaging (DTI), and functional MRI (fMRI), have made it progressively easier to build structural (Alexander-Bloch and Giedd, 2013) or functional (Mancini et al., 2016) brain networks. Among the various types of brain networks, the structural correlation network is built by computing the Pearson's correlation coefficient of structural features (e.g., cortical thickness) between each pair of ROIs across subjects. Its difference from the structural covariance network (SCN), which is often used synonymously, lies in that correlation is normalized by the variance of individual datasets. Therefore, correlations are comparable between datasets of different scales (Alexander-Bloch and Giedd, 2013). Extensive cross-disciplinary precedents suggest that inter-regional structural covariance may result from coordinated neurodevelopment (Raznahan et al., 2011). These synchronized developmental relationships are in turn influenced by physical white matter connections and functional neuronal co-activation, and probably also by other genetic and environmental factors (Alexander-Bloch and Giedd, 2013). Some researchers argued that the structural correlation network may be more akin to the functional network than the white matter fiber structure network (Honey et al., 2010), because the white matter may not be the only source of inter-regional interactions. A longitudinal study of children after birth to 2 years old (Geng X. et al., 2017) shows that functional networks are in place prior to structural networks, hence regional co-activation in functional networks may guide and refine the maturation of SCNs over childhood development. Prominent changes of topological properties take place in the structural covariance/correlation networks since the neonatal stage. Modularity, local efficiency and global efficiency all increase during the first 2 years of life (Fan et al., 2011). After that, development (between the ages of 5 and 18 years) appears to be non-linear, with a substantial but transient shift toward greater integration and less local segregation in late childhood (8–11 years) (Khundrakpam et al., 2013). Primary sensory and motor networks are well-developed in early childhood but expand in early adolescence before pruning, while language, social-emotional, and other cognitive networks are relatively undeveloped in younger age groups and show increasingly distributed topology in older children (Zielinski et al., 2010; Khundrakpam et al., 2013). During the ages of 12–30 years, the network integration continues to increase in the white matter structural connectivity networks (Dennis et al., 2013). The frontal cortex has a disproportionate number of decreases while the temporal cortex has a disproportionate number of increases in fiber density. The lifespan changes of both decreased segregation (within-module connectivity) and increased integration (between-module connectivity) have also been replicated in functional connectivity networks (Chan et al., 2014; Wen et al., 2019; Zhang et al., 2019). A study of SCNs across participants at ages of 8–85 years demonstrates that healthy age-related brain degeneration mirrors development, with the areas of the brain that develop later also degenerating earlier (Douaud et al., 2014). This is supported by another research of eight SCNs corresponding to the well-known functional intrinsic connectivity networks that all the SCNs, except the primary motor network, have distributed topology at young ages (18–23 years), a sharply localized topology at middle ages (30–58 years), and are relatively stable at older ages (61–89 years) (Li et al., 2013). Nevertheless, declines in functional connectivity occur notably later in life than what is reported in the structural connectivity (Lindenberger, 2014). This may suggest that the brain actively maintains patterns of functional interactions for as long as possible (Zuo et al., 2017).

Prior works on the analysis of developmental brain networks generally fall into three categories. (1) The first category (Honey et al., 2010; Vértes and Bullmore, 2015) depicts the developmental curves of network characteristics using different graph-theoretic metrics, such as small-world properties and network efficiency (He and Evans, 2010). This group of methods represents each brain network with one or more topological attributes. (2) The second category investigates the dynamic modular organization along development (Nie et al., 2011; Betzel and Bassett, 2016; Zhang et al., 2018, 2019). Each module is defined as a group of brain regions with dense intra-modular connections and is often related to specific functions. The methods in this category analyze the networks from the respect of intra- or inter-modular connections, but cannot reveal the composition of each connectivity, i.e., the different factors that contribute to the formation of each connectivity. (3) The third category employs many matrix decomposition methods, such as principal component analysis (PCA), independent component analysis (ICA), and non-negative matrix factorization (NMF), to identify the intrinsic components (Ghanbari et al., 2014; Sotiras et al., 2017) or connectivity states (Leonardi et al., 2013; Calhoun et al., 2014; Kopell et al., 2014). Those methods represent each network with either a centroid network or a linear combination of subnetworks. The subnetwork representation is advantageous in preserving detailed connectivity information so that it can reconstruct the developmental networks.

Among the various matrix decomposition methods, NMF is highlighted with its non-negativity constraint on both of the factor matrices (Lee and Seung, 1999). This is an important constraint because it leads to sparse, parts-based representations, which are more interpretable than the non-sparse and global features. Under the NMF framework, we may interpret the decomposed basis matrix as *meta-networks*, where the non-negative elements indicate the strength of connections. Meanwhile, we may interpret the decomposed coefficient matrix of NMF as *covarying trajectories*, whose non-negative elements suggest the contribution of the meta-networks along development. However, although the standard NMF method makes the decomposition results interpretable, there are still at least three other challenges to be addressed in the developmental network analysis.

- *Noise:* Noise contamination is a common problem in brain neuroimaging. Many factors contribute to noise in MRI, such as scanner noise and subject noise. Despite the preprocessing of neuroimages, the constructed developmental structural correlation networks are inevitably affected by noise to some extent. A good developmental network analysis method is expected to exclude, or at least reduce, the impact of noise.
- *Temporal smoothness:* In the existing neural network models, nervous systems can change smoothly by slowly adjusting connectivity strength (Enquist and Ghirlanda, 2005). If the developmental brain networks change smoothly with the elapsing of time, the covarying trajectories of the underlying meta-networks are also expected to evolve smoothly along development. Besides, it is beneficial to enhance the model robustness by considering temporal smoothness.
- *Non-overlapped connections:* Prior works on brain network analysis often produce non-overlapped regions in each subnetwork. However, every brain region evolves, grows and adapts within the whole brain context. It could be misleading if researchers overemphasize the evolution of brain network organization in a modular fashion (Krasnegor, 2013). Instead, since each connection develops in a unique way, it would be biologically more meaningful to produce meta-networks with non-overlapped connections.

In this study, we develop a new mathematical model to deal with all the above challenges. With this model, we decompose five meta-networks and their covarying trajectories from the developmental structural correlation networks across subjects at 3–20 years of age. Analysis of the meta-networks reveals the dynamic negotiation among different factors in brain network development. In particular, three important aspects of normal brain network development are highlighted as follows: (1) two types of indirect connections are gradually replaced by direct connections, (2) the connections with some language-related hub regions (bilateral IFGoperc, see full name in Table 2) peak at the age of ~7 years, (3) the connections with some emotion-related hub regions (ACG.R and MCG.R) peak at the age of 12~13 years.

## Materials and Methods

### Participants

Data used in this article are from the Pediatric MRI Data Repository^1^ released by the NIH MRI Study of Normal Brain Development (Evans and Group, 2006), which is a multi-site study that aims at investigating brain maturation in a normal sample. In this study, we adopt 933 sessions of 445 subjects aging from 3 to 20 years old. No participant had prior history of medical illnesses with CNS implications, IQb70, or intra-uterine exposure to substances known or highly suspected to alter brain structure or functions (Evans and Group, 2006). Several participants are scanned in two or more MRI sessions over a 5–6 year period. In this study, we do not utilize the longitudinal information of the same subject but treat all the sessions independently. To obtain the developmental structural correlation networks, we partition the 933 sessions into 18 groups according to their ages (3~20 years). The age in years are obtained by subtracting the date of birth from the date of visit. Table 1 provides the basic demographic information of the participants and sessions. More detailed gender distribution at each age can be found in Figure S1. As shown in Table 1 and Figure S1, males and females have close distribution at each age.

**Table 1:** Summary of the basic demographic information of the participants.

	**Male**	**Female**	**Total**
Participants	214	231	445
Sessions	437	496	933
Session Age (years)	11.4 ± 4.3	11.5 ± 4.3	11.5 ± 4.3
Age range (years)	3~20	3~20	3~20

### Data Preprocessing

For each T1-weighted MR image, we first perform skull stripping to remove non-cerebral tissues, the cerebellum and brain stem with BET (Smith, 2002) in FSL (version 4.3). Then, each brain image is segmented into gray matter (GM), white matter (WM), and cerebrospinal fluid (CSF) regions (Zhang et al., 2001) with FAST in FSL (version 4.3). Next, we reconstruct inner and outer cortical surfaces represented by triangular meshes (Liu et al., 2008). After registration with a high-dimensional non-linear hybrid volumetric/surface registration method (Liu et al., 2004), we have each cortical surface parcellated into 78 regions based on the automated anatomical labeling template (Tzourio-Mazoyer et al., 2002). Table 2 summarizes the 78 cortical surface regions of interest.

**Table 2:** Seventy eight cortical regions of automated anatomical labeling template.

**Abbreviation**	**Region**	**Abbreviation**	**Region**
Precentral gyrus left	PreCG.L	Precentral gyrus right	PreCG.R
Superior frontal gyrus left, dorsolateral	SFGdor.L	Superior frontal gyrus right, dorsolateral	SFGdor.R
Superior frontal gyrus left, orbital part	ORBsup.L	Superior frontal gyrus right, orbital part	ORBsup.R
Middle frontal gyrus left	MFG.L	Middle frontal gyrus right	MFG.R
Middle frontal gyrus left, orbital part	ORBmid.L	Middle frontal gyrus right, orbital part	ORBmid.R
Inferior frontal gyrus left, opercular part	IFGoperc.L	Inferior frontal gyrus right, opercular part	IFGoperc.R
Inferior frontal gyrus left, triangular part	IFGtriang.L	Inferior frontal gyrus right, triangular part	IFGtriang.R
Inferior frontal gyrus left, orbital part	ORBinf.L	Inferior frontal gyrus right, orbital part	ORBinf.R
Rolandic operculum left	ROL.L	Rolandic operculum right	ROL.R
Supplementary motor area left	SMA.L	Supplementary motor area right	SMA.R
Olfactory cortex left	OLF.L	Olfactory cortex right	OLF.R
Superior frontal gyrus left, medial	SFGmed.L	Superior frontal gyrus right, medial	SFGmed.R
Superior frontal gyrus left, medial orbital	ORBsupmed.L	Superior frontal gyrus right, medial orbital	ORBsupmed.R
Gyrus rectus left	REC.L	Gyrus rectus right	REC.R
Insula left	INS.L	Insula right	INS.R
Anterior cingulate gyri left	ACG.L	Anterior cingulate gyri right	ACG.R
Median cingulate gyri left	MCG.L	Median cingulate gyri right	MCG.R
Posterior cingulate gyrus left	PCG.L	Posterior cingulate gyrus right	PCG.R
Parahippocampal gyrus left	PHG.L	Parahippocampal gyrus right	PHG.R
Calcarine cortex left	CAL.L	Calcarine cortex right	CAL.R
Cuneus left	CUN.L	Cuneus right	CUN.R
Lingual gyrus left	LING.L	Lingual gyrus right	LING.R
Superior occipital gyrus left	SOG.L	Superior occipital gyrus right	SOG.R
Middle occipital gyrus left	MOG.L	Middle occipital gyrus right	MOG.R
Inferior occipital gyrus left	IOG.L	Inferior occipital gyrus right	IOG.R
Fusiform gyrus left	FFG.L	Fusiform gyrus right	FFG.R
Postcentral gyrus left	PoCG.L	Postcentral gyrus right	PoCG.R
Superior parietal gyrus left	SPG.L	Superior parietal gyrus right	SPG.R
Inferior parietal left	IPL.L	Inferior parietal right	IPL.R
Supramarginal gyrus left	SMG.L	Supramarginal gyrus right	SMG.R
Angular gyrus left	ANG.L	Angular gyrus right	ANG.R
Precuneus left	PCUN.L	Precuneus right	PCUN.R
Paracentral lobule left	PCL.L	Paracentral lobule right	PCL.R
Heschl gyrus left	HES.L	Heschl gyrus right	HES.R
Superior temporal gyrus left	STG.L	Superior temporal gyrus right	STG.R
Temporal pole left superior gyrus	TPOsup.L	Temporal pole right superior gyrus	TPOsup.R
Middle temporal gyrus left	MTG.L	Middle temporal gyrus right	MTG.R
Temporal pole left middle gyrus	TPOmid.L	Temporal pole right middle gyrus	TPOmid.R
Inferior temporal gyrus left	ITG.L	Inferior temporal gyrus right	ITG.R

The cortical thickness is measured in the native space using the shortest distance at each vertex (Fischl and Dale, 2000; Li et al., 2012). The regional cortical thickness is computed as the average thickness of all the vertices belonging to the same ROI. To remove the effects of multiple confounding variables (including gender and the whole-brain mean cortical thickness), a linear regression analysis is performed at every cortical region for each age (He et al., 2007). Then, the residual of regression is taken as the final cortical thickness value. We treat the sessions of the same age equally, and compute the pairwise similarity of cortical regions with the inter-regional Pearson's correlation across subjects at each age (Alexander-Bloch and Giedd, 2013). Take age 9 for example; there are 76 different subjects of 9 years of age, then each ROI is represented with a cortical thickness vector of length 76. By computing the pairwise Pearson's correlation coefficient among different ROI vectors, we build a 78 × 78 inter-regional correlation network for age 9. This study is performed on the basis of our previous work (Nie et al., 2013). For more detailed information about network construction, please refer to the literature (Nie et al., 2013). We use the absolute values in each network (Khundrakpam et al., 2013) so that each entry represents the strength of the pairwise inter-regional correlation. In this way, we obtain 18 non-negative cortical thickness correlation networks with equal size (78 × 78) from the 3 year-old children to the 20 year-old adults (Figure S2).

### Mathematical Model for Meta-Network Analysis

**Figure 1:**
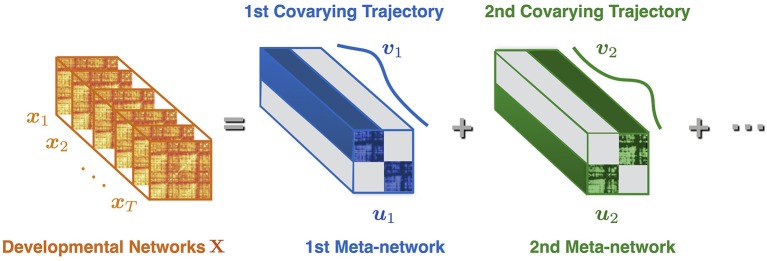
Illustration of meta-network decomposition. The developmental networks **X** = [***x***_1_, ***x***_2_, ⋯, ***x***_*T*_] are a sequence of brain networks at different ages (indexed from 1 to *T*). The meta-network decomposition method decomposes **X** into a small number of non-overlapped meta-networks **U** = [***u***_1_, ***u***_2_, ⋯], whose dynamic weight over time is indicated by the covarying trajectories **V** = [***v***_1_, ***v***_2_, ⋯]. Each meta-network (e.g., ***u***_1_) represents a distinctive network connection pattern. Each covarying trajectory (e.g., ***v***_1_) reveals the dynamic contribution of the corresponding meta-network with the passing of time.

Suppose **X** = [***x***_1_, ***x***_2_, ⋯, ***x***_*T*_] is the sequence of developmental brain networks from age 3 to 20 years, $x_{i}\in {\mathbb{R}}^{n}$ is the *i*^*th*^ vectorized network of length *n* = 78 × (78–1)/2, and *T* is the number of time points (or networks). Meta-network decomposition aims at uncovering the intrinsic meta-networks **U** = [***u***_1_, ***u***_2_, ⋯, ***u***_*r*_] and their corresponding covarying trajectories **V** = [***v***_1_, ***v***_2_, ⋯, ***v***_*r*_] (Figure 1). Here $u_{j}\in {\mathbb{R}}^{n}$ is the *j*^*th*^ vectorized meta-network, *r* is the number of meta-networks, $v_{j}\in {\mathbb{R}}^{T}$ is the covarying trajectory of ***u***_*j*_, indicating the contribution of ***u***_*j*_ over time.

To deal with the first challenge of noise interference, we assume that the observed networks (**X**) are composed of the true brain networks (**X**_0_) and arbitrary noise (**E**) two parts. Since true signals are often of, or can be well approximated by, a low rank structure (Markovsky, 2011), we impose the low rank constraint on **X**_0_. Formally written,

(1) $$\begin{array}{r} \text{X}={\text{X}}_{0}+\text{E},\;\text{rank}\left({\;\text{X}}_{0}\right)\leq r \end{array}$$

Meanwhile, we incorporate the non-negativity constraints of the NMF model to ensure the interpretability of results, leading to the following objective function.

(2) $$\begin{array}{r} \min_{\text{U},\text{V}\geq 0}||\text{X}-\text{U}{\text{V}}^{T}|{|}_{F}^{2}+2\lambda ||\text{U}{\text{V}}^{T}\;|{|}_{{\;}^{*}} \end{array}$$

The first term of Equation (2) is the approximation error of ||**X**−**X**_0_||, the second term is a regularization term that penalizes the rank of **X**_0_. Since it has been proved (Srebro and Shraibman, 2005) that minimizing the nuclear norm of two non-negative matrix products is equivalent to minimizing their Frobenius norm, i.e., $\min2\lambda ||\;\text{U}{\text{V}}^{T}|{|}_{{}^{*}}=\min\lambda \left(||\text{U}|{|}_{F}^{2}+||\text{V}|{|}_{F}^{2}\right)$, we can replace the nuclear norm in Equation (2) and obtain the following objective function.

(3) $$\begin{array}{r} {\text{min}}_{\text{U},\text{V}\geq 0}||\text{X}-\text{U}{\text{V}}^{T}|{|}_{F}^{2}+\lambda \left(||\text{U}|{|}_{F}^{2}+||\text{V}\;|{|}_{F}^{2}\right) \end{array}$$

To address the second challenge of temporal smoothness, we introduce a regularization term to evaluate the smoothness of the covarying trajectories **V**.

(4) $$\begin{array}{r} {\text{min}}_{\text{U},\text{V}\geq 0}||\text{X}-\text{U}{\text{V}}^{T}|{|}_{F}^{2}+\lambda \left(||\text{U}|{|}_{F}^{2}+||\text{V}|{|}_{F}^{2}\right)+\beta \text{tr}\left({\text{V}}^{T}\text{L}\text{V}\;\right) \end{array}$$

In Equation (4), the third term $tr\left({\text{V}}^{T}\text{L}\text{V}\right)=\sum_{i,j=1}^{T}w_{ij}||v_{i\cdot }-v_{j\cdot }|{|}^{2}$ regularizes the temporal smoothness of **V**, where **L** is the Laplacian matrix, $w_{ij}=e^{-||x_{i}-x_{j}||/2\sigma^{2}}$ measures the similarity between the networks at the *i*^*th*^ and *j*^*th*^ time points, ***v***_*i*_ = [*v*_*i*1_, *v*_*i*2_, ⋯ , *v*_*ir*_ ] is the *i*^*th*^ row of **V**, which records the weight of the meta-networks at the *i*^*th*^ time point. Hence, if the developmental networks change smoothly with the passing of time, the decomposed covarying trajectories will also move smoothly over time.

Finally, we enforce an orthogonality constraint on **U** to produce non-overlapped meta-networks. With this constraint (**U**^*T*^**U** = **I**), each connection will be grouped with other covarying connections into a unique meta-network.

(5) $$\begin{array}{r} {\text{min}}_{\text{U},\text{V}\geq 0}||\;\text{X}-\text{U}{\text{V}}^{T}|{|}_{F}^{2}+\lambda \left(||\text{U}|{|}_{F}^{2}+||\text{V}|{|}_{F}^{2}\right)+\beta \text{tr}\left({\text{V}}^{T}\text{L}\text{V}\right)\\ s.t.\;{\text{U}}^{T}\text{U}={\text{I}}_{r\;} \end{array}$$

Note that in Equation (5), the Frobenius norm of **U** is fixed under the orthogonality constraint ($||\text{U}|{|}_{F}^{2}=tr\left({\text{U}}^{T}\text{U}\right)=r$). Therefore, the final objective function can be written in the following concise form.

(6) $$\begin{array}{r} {\text{min}}_{\text{U},\text{V}\geq 0}||\text{X}-\text{U}{\text{V}}^{T}|{|}_{F}^{2}+\lambda \left(||\text{V}|{|}_{F}^{2}\right)+\beta \text{tr}\left({\text{V}}^{T}\text{L}\text{V}\right)\\ s.t.\;{\text{U}}^{T}\text{U}={\text{I}}_{r} \end{array}$$

It can be proved that the multiplicative updating rules for **U** and **V** are, respectively, as follows.

(7) $$\begin{array}{r} \text{U}\leftarrow \text{U}⊙\frac{\text{X}\text{V}}{\text{U}{\text{U}}^{T}\text{X}\text{V}} \end{array}$$

(8) $$\begin{array}{r} \text{V}\leftarrow \text{V}⊙\frac{{\text{X}}^{T}\text{U}+\beta \text{W}\text{V}}{\text{V}{\text{U}}^{T}\text{U}+\lambda \text{V}+\beta \text{D}\text{V}} \end{array}$$

The symbol ⊙ represents the element-wise product, and the division symbol is also element-wise. The proof of convergence of the multiplicative updating rule for **U** and **V** is similar to that of Lee and Seung (1999). To avoid local minimum, we adopt an initialization strategy on **U** and **V** similar to that of the *kmeans* algorithm, i.e., repeating multiple (100) times with random initializations and choosing the best one. The proposed method is robust to a wide range of the regularization parameters (Figure S3).

### Model Selection

We determine the number of meta-networks by examining the reproducibility of the decomposed meta-networks and covarying trajectories in a split-age setting. First, we divide the developmental brain networks into two halves with odd-numbered ages (3, 5, 7, 9, 11, 13, 15, 17, and 19 years) and even-numbered ages (4, 6, 8, 10, 12, 14, 16, 18, and 20 years). Then we quantify the reproducibility by computing the cosine similarity between the meta-networks (or covarying trajectories) of the two splits after matching them with the Hungarian algorithm (Kuhn, 2005), as done in Lange et al. (2004). The cosine similarity is advantageous in evaluating the covarying trajectory reproducibility, because it is a judgment of orientation instead of magnitude.

To verify the reliability of the decomposition results, we also examine the reconstruction error with the increase of meta-network number. The evaluation criterion is root mean square error (RMSE),

(9) $$\begin{array}{r} \text{RMSE}\left(r\right)=\;\frac{||\;\text{X}-{\text{U}}_{\text{r}}{\text{V}}_{\text{r}}^{T}|{|}_{\text{F}}}{\sqrt{\text{nT}}},\forall \text{r}=1,\cdots \;,T \end{array}$$

where *nT* is the number of connections in **X**. On the basis of RMSE, we further compute the improvement of RMSE contributed by each meta-network, i.e., Δ*RMSE*(*r*) = *RMSE*(*r*)−*RMSE*(*r*−1), where $RMSE\left(0\right)=\frac{||\;\text{X}\;|{|}_{F}}{\sqrt{nT}}$.

After the determination of the meta-network number, we examine the ratios of the reconstructed networks (${\text{X}}_{0}=\text{U}{\text{V}}^{T}$) and the noise networks (**E**) in the developmental networks (**X**). For the *j*^*th*^(∀*j*∈[1, *T*]) age, we compute the ratios by dividing the total connection weight of the reconstructed network and in the noise network by that in the developmental network.

(10) $$\begin{array}{r} r_{{\text{X}}_{0}}\left(j\right)=\;\frac{\sum_{i=1}^{n}{\text{X}}_{0}\left(i,j\right)}{\sum_{i=1}^{n}\text{X}\left(i,j\right)},r_{\text{E}}\left(j\right)=\;\frac{\overset{n}{\underset{i=1}{\sum }}\text{E}\left(i,j\right)}{\overset{n}{\underset{i=1}{\sum }}\text{X}\left(i,j\right)} \end{array}$$

In Equation (10), *r*_**X**_0__(*j*) and *r*_**E**_(*j*) respectively denote the ratios of the reconstructed network and the noise network in the developmental network at the *j*^*th*^ age. Note that **X** = **X**_0_ + **E**, hence *r*_**X**_0__(*j*)+*r*_**E**_(*j*) = 1. We further compute the mean ratio of the reconstructed networks and the noise networks by averaging *r*_**X**_0__(*j*) and *r*_**E**_(*j*) over different ages.

(11) $$\begin{array}{r} \overset{\bar{\;}}{r_{{\text{X}}_{0}}}=\frac{1}{T}\overset{T}{\underset{j=1}{\sum }}r_{{\text{X}}_{0}}\left(j\right),\;\bar{r_{\text{E}}}=\frac{1}{T}\overset{T}{\underset{j=1}{\sum }}r_{\text{E}}\left(j\right) \end{array}$$

## Results

We apply our method to the developmental structural correlation networks at 3–20 years of age and obtain five meta-networks as well as covarying trajectories.

### Reproducibility and Reconstruction

**Figure 2:**
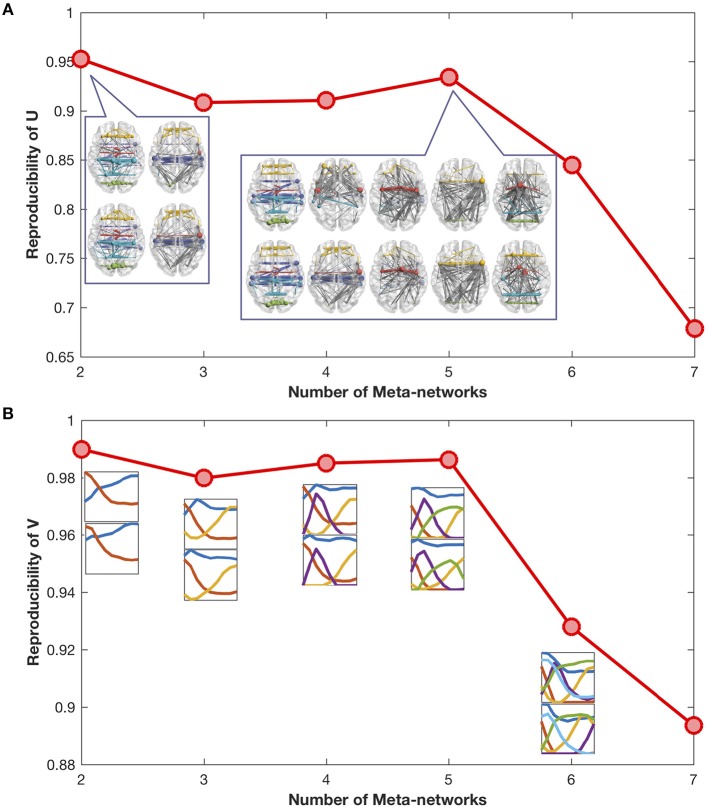
**(A)** Reproducibility of meta-networks with the increase of meta-networks. The meta-networks decomposed from the odd-number aged developmental networks (in upper row) are similar to the meta-networks from the even-number aged developmental networks (in lower row). The maximal reproducibility is 1, the minimal reproducibility is 0. **(B)** Reproducibility of covarying trajectories with the increase of meta-networks. The covarying trajectories of the odd-number aged developmental networks (in upper row) are similar to the covarying trajectories of the even-number aged developmental networks (in lower row).

The reproducibility of meta-networks and covarying trajectories generally declines with the increase of meta-networks (Figures 2A,B). Yet, there is a clear peak at the meta-network number of 5, which achieves a good balance between model expressiveness and result reproducibility. Therefore, in this study we set the meta-network number as 5.

**Figure 3:**
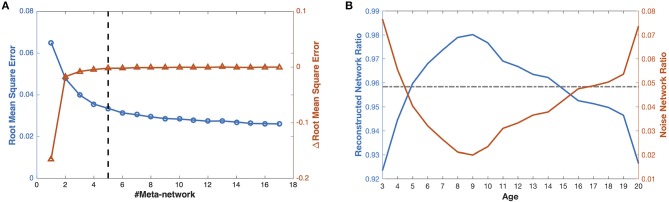
The reconstruction of the developmental structural correlation networks. **(A)** Root mean square error of increasing number of meta-networks. The left Y axis for blue circles represents the root mean square error (RMSE), the right Y axis for red triangles represents the improvement of RMSE (ΔRMSE) introduced by each meta-network. **(B)** When the number of meta-networks is 5, the ratios of the reconstructed networks (blue line) and the noise networks (red line) in the developmental networks change with ages. The gray line indicates the mean ratio of the reconstructed networks (in the left blue axis) and the noise networks (in the right red axis) over different ages.

The reconstruction error curves of the decomposed meta-networks and covarying trajectories verify the reliability of our selected meta-network number (Figure 3A). On one side, the RMSE curve gradually declines with the increase of meta-networks. On the other side, the ΔRMSE curve progressively converges to 0, which indicates the decreasing contribution of the meta-networks in network reconstruction. When there are 5 meta-networks, ΔRMSE is already sufficiently small (≤0.002) that introducing additional meta-networks can hardly make significant improvement. Therefore, it is reasonable to set the meta-network number to 5 in this study.

With 5 meta-networks, the reconstructed networks and the noise networks, respectively, account for 96 and 4% of the developmental networks on average (Figure 3B). As the age grows, the ratios of the reconstructed networks first climb (>98%) and then decline. In contrast, the ratios of the noise networks change in the opposite direction. Moreover, a statistical *t*-test demonstrates that the ratios of the noise networks are negatively correlated with the session numbers at different ages (Figure S1) (*r* = −0.66, *p* < 0.002, one tailed), which indicates that a larger number of sessions leads to smaller noise.

### Meta-Networks 1–3: Direct vs. Indirect Connections

**Figure 4:**
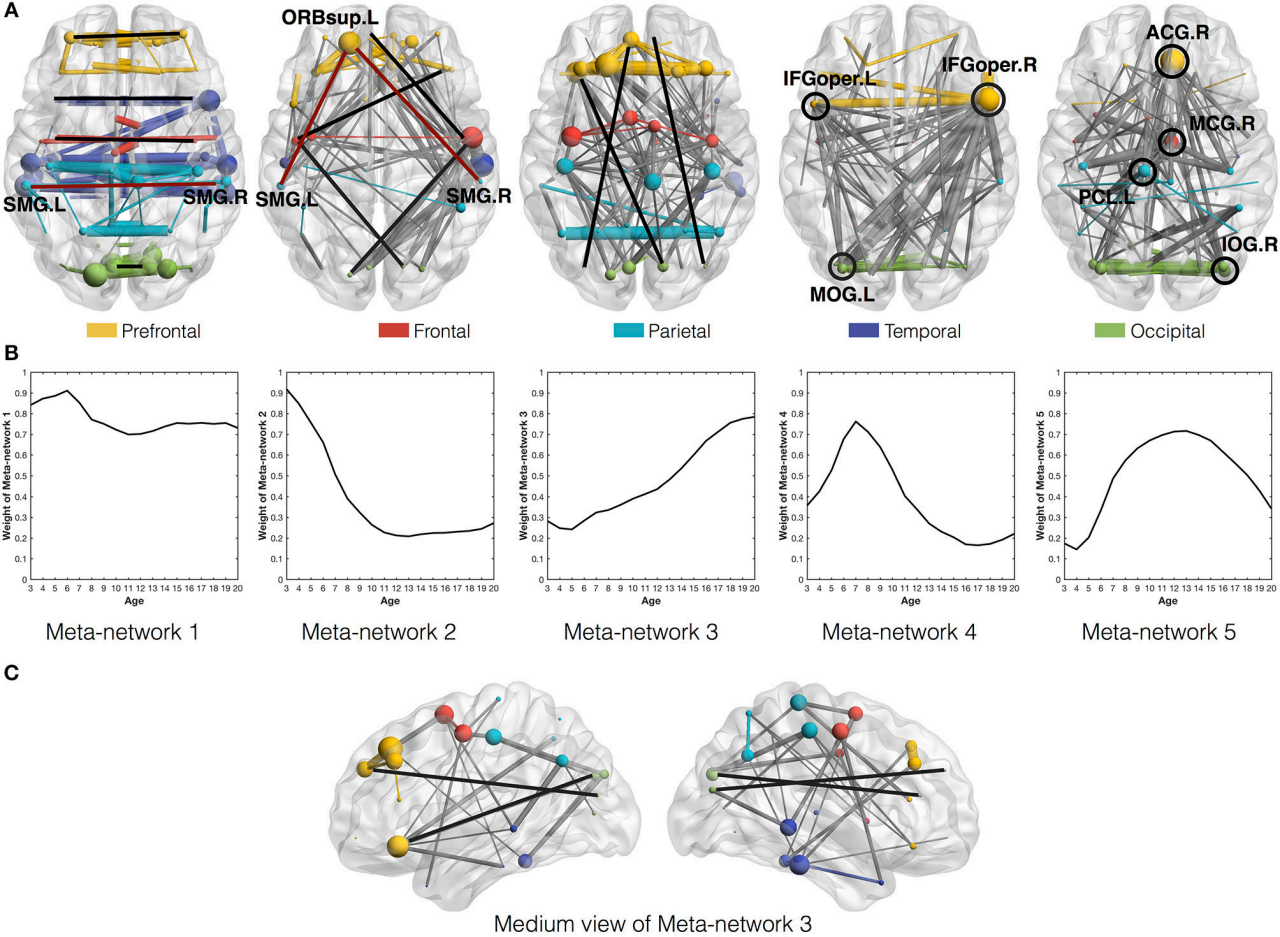
**(A)** Axial view of the five meta-networks depicted with BrainNet Viewer (Xia et al., 2013). The size of each node (i.e., ROI) is proportional to its degree in the meta-network. The width of the edge is proportional to its correlation strength. Different ROIs are rendered with different colors according to their anatomical locations as suggested by previous studies (Wang et al., 2007). The intra-modular edges are assigned with the same colors as their linked nodes, while the inter-modular edges are colored in gray. The featured connections in each meta-network are highlighted in red/black lines and circles. Specifically, the red lines in the first and second meta-networks illustrate the direct vs. indirect connections between homotopic regions in the two hemispheres. The black lines in the second and third meta-networks illustrate the indirect vs. direct connections between the prefrontal and occipital regions. The black circles in the fourth and fifth meta-networks highlight their significant hub regions. **(B)** The covarying trajectories of the five meta-networks move smoothly with the growth of age. **(C)** The medium view of Meta-network 3 presents a clearer illustration of the direct connections between prefrontal and occipital regions within the same hemispheres.

Among the five meta-networks, the first three meta-networks show a tradeoff between the direct and indirect connection patterns (Figure 4). While Meta-network 1 is dominant with direct connections between homotopic regions of two hemispheres (Figure 4A), Meta-network 3 is featured with long-distance direct connections between the prefrontal and occipital regions (Figures 4A,C). In contrast, Meta-network 2 is highlighted with the corresponding two types of indirect connections. One is the indirect connections between homotopic regions through prefrontal areas (shaped like “∧” in Figure 4A). Another is the indirect connections between the prefrontal and occipital regions through frontal/temporal areas (shaped like 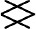. A typical example is the direct connection between bilateral supramarginal gyri (SMG.L and SMG.R) in Meta-network 1 vs. the corresponding indirect connection through the orbital part of the left superior frontal gyrus (ORBsup.L) in Meta-network 2 (Figure 4A). The quantitative validation of the connection patterns in the first three meta-networks are illustrated in Figures S4A–D.

**Figure 5:**
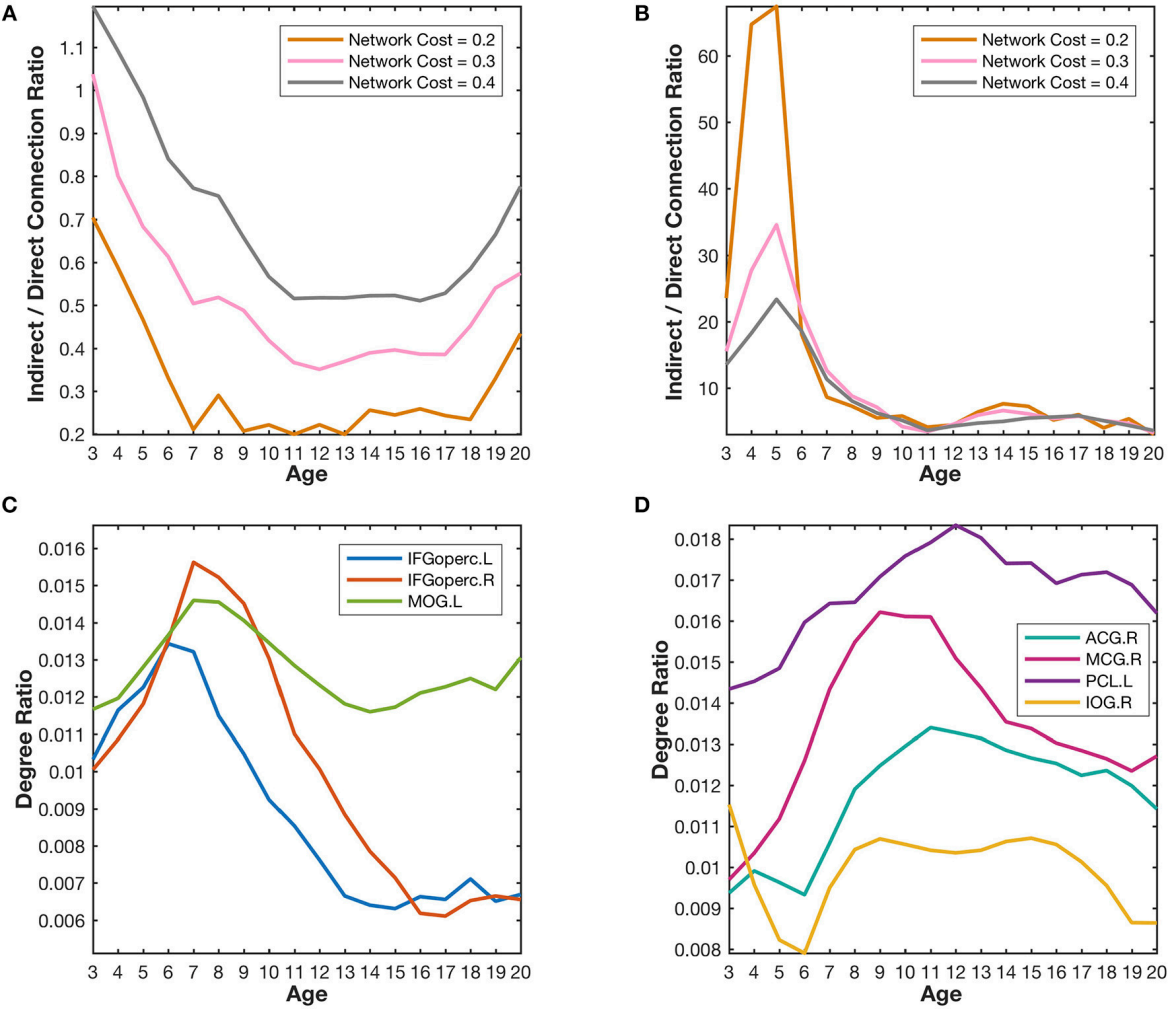
Quantitative validation of the major findings in the developmental networks. **(A)** The indirect/direct connection ratio between the homotopic parietal regions in two hemispheres generally declines with the growth of age. The network cost refers to the ratio of the number of reserved edges in a network to the maximum possible number of pair-wise connections (78 × 77/2). Lower network cost leads to stronger reserved correlations. **(B)** The indirect/direct connection ratio between the prefrontal and occipital regions generally declines with the growth of age. **(C)** The degree ratios of the language-related regions (IFGoperc.L, IFGoperc.R, and MOG.L) increase from the age of 3 years to the age of 7 years and then declines quickly. The developmental trajectories of those identified hub regions are very similar to the covarying trajectory of the fourth meta-network. **(D)** The degree ratios of the emotion-related regions (ACG.R, MCG.R, PCL.L, and IOG.R) reach their peaks at the age of ~12 years and decrease slowly after that. The developmental trajectories of those emotion-related hub regions are very similar to the covarying trajectory of the fifth meta-network.

Combined with the covarying trajectories of the first three meta-networks (Figure 4B), we find that the indirect connection patterns are gradually replaced by the direct connection patterns with the growth of age. On one side, the weight of the direct connection patterns either stays high (in Meta-network 1) or increases progressively (in Meta-network 3). On the other side, the weight of the indirect connection patterns (in Meta-network 2), although starting from the highest value at the age of 3 years, declines continuously until 13 years of age and remains stable afterward. This observation is also quantitatively validated in the developmental networks (Figures 5A,B).

### Meta-Networks 4–5: Hub Structure

**Figure 6:**
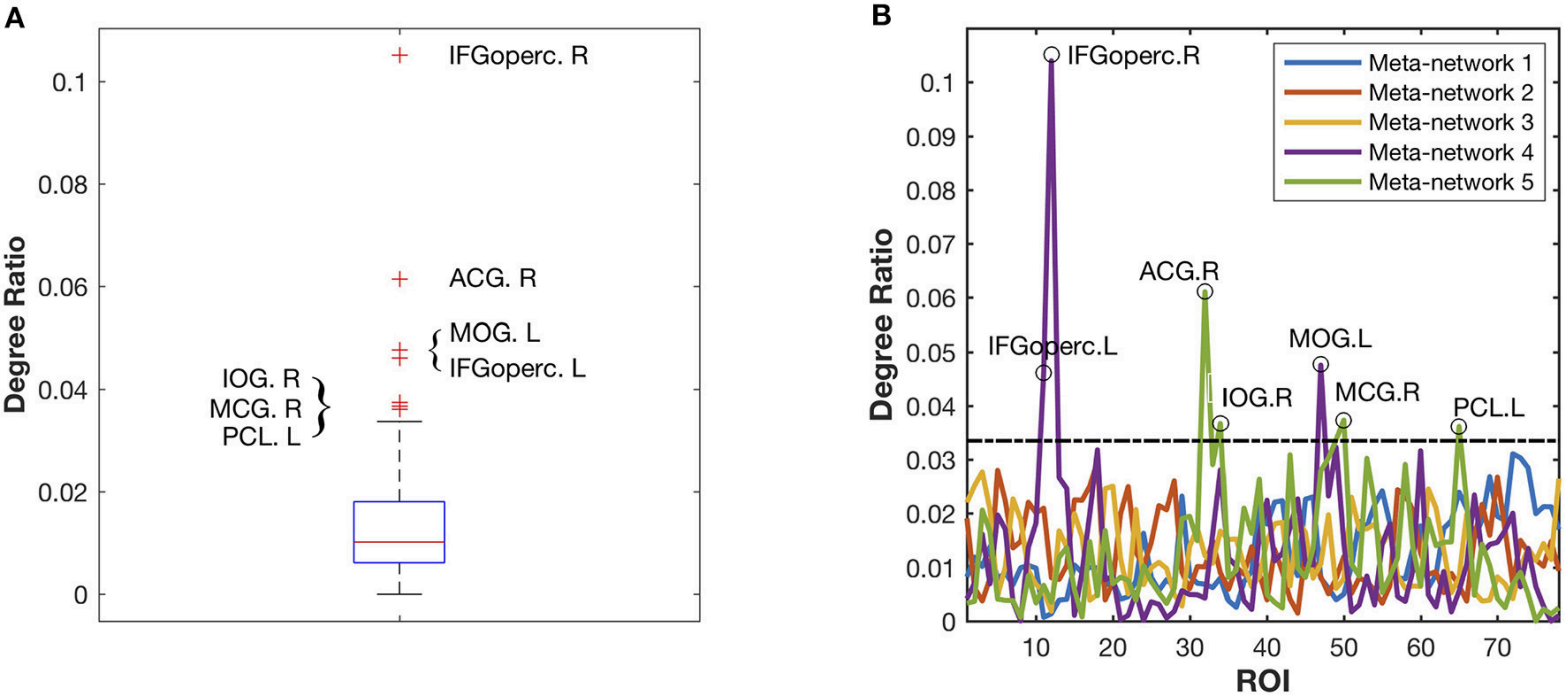
Identification of the significant hub regions from the five meta-networks. **(A)** The box plot of all the degree ratios of the 78 ROIs in the five meta-networks. Seven significant hub regions are identified as outliers (beyond the whiskers) from the five meta-networks. **(B)** The degree ratio distribution of the 78 ROIs in the five meta-networks. The black circles highlight the identified seven hub regions, which have significantly higher degree ratios than the others. Among them, three hub regions including IFGoperc.L, IFGoperc.R, and MOG.L are in Meta-network 4, while the other four hub regions, including ACG.R, MCG.R, IOG.R, and PCL.L, are in Meta-network 5.

Different from the previous three meta-networks, the fourth and fifth meta-networks are characterized with distinctive hub structures (Figure 4A). Seven significant hubs are identified from the five meta-networks depicted in a box plot (Figure 6). Three of them lie in Meta-network 4, including the bilateral opercular inferior frontal gyrus (IFGoperc.R and IFGoperc.L) and left middle occipital gyrus (MOG.L) (Figure 4A). Meta-network 5 contains the other four significant hub regions, including the right anterior cingulate gyrus (ACG.R), right middle cingulate gyrus (MCG.R), left paracentral lobule (PCL.L), and right inferior occipital gyrus (IOG.R) (Figure 4A). The quantitative validation of the connection patterns in the fourth and fifth meta-networks are illustrated in Figures S4E,F. We also note that the hub regions in the fourth and fifth meta-networks are densely connected with each other. The quantitative analysis of weighted rich club coefficients (see Supplementary Method Rich-Club Structure) statistically demonstrates the significance of rich clubs (Van Den Heuvel and Sporns, 2011) in the fourth and fifth meta-networks (Figure S6).

The (weighted) covarying trajectories of the fourth and fifth meta-networks reveal the degree ratio development of their hub regions in the developmental networks (see Supplementary Method Covarying Trajectory and Degree Ratio). For the fourth meta-network, its covarying trajectory quickly climbs up until 7 years of age and then drops sharply. As to the fifth meta-network, its covarying trajectory starts to rise from the age of 3 years, reaches its peak at the age of 12~13 years and then declines gradually. The quantitative analysis of the developmental networks demonstrates that the development trajectories of the identified hub regions (Figures 5C,D) are very similar to the (weighted) covarying trajectories of the fourth and fifth meta-networks (Figure S5).

## Discussion

We develop a novel meta-network decomposition method to provide a dynamic view of how the different underlying meta-network patterns negotiate with each other during the development of brain networks. Compared with the existing developmental brain network analysis methods, such as graph-theoretical approaches (Honey et al., 2010; Vértes and Bullmore, 2015), modular organization methods (Nie et al., 2011; Betzel and Bassett, 2016) and matrix decomposition methods (Leonardi et al., 2013; Calhoun et al., 2014; Ghanbari et al., 2014; Kopell et al., 2014; Sotiras et al., 2017), our proposed method is advantageous in several ways. First, it is not only interpretable with non-negative decomposition results, but also robust to noise interference due to the low rank constraint. Second, it produces smooth covarying trajectories according to the smoothness of the evolution of the developmental networks. This agrees with the existing neural network models that nervous systems can change smoothly by slowly changing connectivity strength (Enquist and Ghirlanda, 2005). Third, it views connections, instead of regions, as the basic elements of a network and groups the covarying connections into non-overlapped meta-networks. Therefore, all the inter-regional brain connections are well-preserved for unbiased analysis. Except from the developmental structural correlation brain networks, our method is also applicable to other imaging modalities, such as fMRI (Calhoun et al., 2001; Beckmann and Smith, 2005; Esposito et al., 2005; Wu et al., 2017) and fNIRS (functional near-infrared spectroscopy) (Geng S. et al., 2017). As long as the spatiotemporal imaging features can be organized into longitudinal brain networks (e.g., by computing pairwise correlations within a time bin), the proposed method can be universally applied to uncover the underlying meta-networks and quantify the change in their contribution with the passing of time.

In this study, we use the proposed method to uncover five distinctive meta-networks from the cortical-thickness correlation networks for 3–20 years of age. These five meta-networks are generally categorized into two groups. The first group, composed of the first three meta-networks, reflect the gradual replacement of two types of indirect connections by direct ones along development. The direct correlation of cortical thickness across participants may indicate the direct synaptic connections, while the indirect correlation may indicate the polysynaptic connections between spatially distributed regions that are separated by the same physical distance. Since direct neural connections are generally believed to use less time for signal transmission than the polysynaptic connections (Grossenbacher, 2001), the replacement of indirect connections by direct ones may suggest the increase of network global efficiency in the normal brain development (Achard and Bullmore, 2007; Vogel et al., 2010; Bullmore and Sporns, 2012). This agrees with the research of Vogel et al. (2010) that regional interactions change from being predominantly anatomically local in children to interactions spanning longer cortical distances in young adults.

The second group of meta-networks, including the fourth and fifth meta-networks, are characterized with significant hub regions and rich club structures. The hubs of the fourth meta-network include bilateral IFGoperc. and MOG.L. It has been widely accepted that the opercular inferior frontal gyri play a crucial role in language production, such as speech intonation, word generation, linguistic fluency, grammar and sentence comprehension (Friederici et al., 2003; Amunts et al., 2004). The degree ratio of bilateral IFGoperc. in the fourth meta-network is about a factor of eight of their degree ratio (2 × 1/78) in a random network with the same number of nodes (Figure S4E). MOG.R, as the secondary visual cortex, is associated with visual-related functions, such as visuo-spatial information processing (Lamm et al., 2001; Waberski et al., 2008) and visual priming (Slotnick and Schacter, 2004). In recent years, MOG.R has also been observed to be active in confrontation naming, which involves word retrieval processes (Ghosh et al., 2010). Therefore, the fourth meta-network may suggest the connection patterns related with some language functions. The covarying trajectory of the fourth meta-network peaks at the age of ~7 years, which is consistent with the critical period for language acquisition by environment exposure (Hurford, 1991; Purves et al., 2001). We note that in the previous studies, researchers found expanded distribution of structural covariance (Zielinski et al., 2010) and functional connectivity (Koyama et al., 2011) relevant to the language development. Nevertheless, they are not contradictory to our findings, because our study reveals the change of degree ratio of the language-related regions in the developmental structural correlation networks. It is possible when the connections with IFGoperc. increase, the connections with other brain regions may increase more. Therefore, our study to some extent reveals the specialization of the brain network during development.

On the other hand, the hubs of the fifth meta-network include ACG.R, MCG.R, PCL.L, and IOG.R. Among them, ACG.R and MCG.R at the right cingulate gyrus both belong to the limbic system. They are believed to be involved in emotion formation and processing, decision-making, socially-driven interactions and learning (Bush et al., 2000; Hadland et al., 2003; Apps et al., 2013). The degree ratio of ACG.R and MCG.R in the fifth meta-network is about a factor of five of their degree ratio (2 × 1/78) in a random network with the same number of nodes (Figure S4F). Executive control is also found to be related to the anterior cingulate gyrus to suppress inappropriate unconscious priming (Lavin et al., 2013). That may explain why PCL.L, whose neurons are concerned with motor and sensory innervations (Arslan, 2014), is the third hub of the fifth meta-network, because PCL.L may provide auxiliary coordination with the cingulate gyrus in behavior control (Sarkheil et al., 2013). Additionally, the fourth hub IOG.R is important in visual information processing (Rossion et al., 2003; Slotnick and Schacter, 2004; Waberski et al., 2008), which may also provide auxiliary coordination. Therefore, the fifth meta-network may suggest the connection patterns related with emotion function. The corresponding covarying trajectory of the fifth meta-network reaches its peak during adolescence (at the age of 12~13 years). This is supported by the neurobehavioral research that, around the age of 12 years, adolescents begin to show the capacity for visualization of potential outcomes and logical understanding of cause and effect (Steinberg, 2005; Arain et al., 2013).

To show the consistency among the decomposition results on different cortical measures, we further apply the proposed method onto the developmental cortical-curvature correlation networks of the same subjects at 3–20 years of age (Nie et al., 2013). Five cortical-curvature correlation meta-network as well as their corresponding covarying trajectories (Figure S7) are decomposed in comparison with the same number of cortical-thickness correlation meta-networks and their covarying trajectories (Figure 4). Despite of the difference in the two sets of decomposition results due to the different developmental networks, they both show a trend of gradual replacement of indirect connections by direct ones. Therefore, the decomposition results on the developmental cortical-curvature correlation networks further validate the increase of network global efficiency in normal brain development (Achard and Bullmore, 2007; Vogel et al., 2010),(Bullmore and Sporns, 2012).

Compared with other matrix decomposition methods such as the state-of-the-art PCA method, our model imposes additional constraints, especially the non-negativity constraints, on the meta-networks and covarying trajectories. As a result, the proposed model is advantageous in interpreting the covarying trajectories as the dynamic weight of meta-networks along development. Nevertheless, the non-negativity constraints also lead to the loss of negative correlation patterns in meta-networks, but only preserve their strength instead. In respect of reproducibility, our proposed model produces more reproducible meta-networks (or components) than PCA when the meta-network number is between 3 and 5 (Figure S8A). Meanwhile, it also produces more reproducible covarying trajectories (or coefficients) than PCA despite of the change of meta-network number (Figure S8B). Another advantage of our method lies in its smoother covarying trajectories (Figure S8C). Even without the temporal smoothness constraint (β = 0), the covarying trajectories of our proposed method are still much smoother than those of PCA. That may indicate higher robustness of the proposed method against the influence of noise than PCA.

Last but not least, since the meta-networks are determined based on the covarying development of the connections between ROIs, they are actually more complex than the dominant connection patterns. For instance, except from the long direct connections between the prefrontal and occipital regions, there are also some indirect connections in the third meta-network (Figure 4A). Aside from the emotion-related hub regions, there are also some regions underlying different functions in the fifth meta-network. This is reasonable because not all the indirect connections are replaced by the direct connections, and the enhancement of emotion function may require the coordination from auxiliary brain regions. Our interpretation of the discovered meta-networks only reflects the general trend of the underlying tradeoff (Figure 5), but they may mean more than that.

Clearly, one can apply this method to group comparison. The most intuitive way is to directly compare the meta-network patterns between the normal control and patient groups. Alternatively, we can also set the meta-networks as those of the normal control group, and then find out the difference in the covarying trajectories between the normal control and patient groups. The critical time neurodevelopmental diseases, such as autism spectrum disorder (ASD), can be identified in this way. In addition, with the progressively easier collection of DTI or fMRI data, one can also apply the proposed method to analyze the longitudinal brain networks for different subjects. The comparison of the meta-networks (and covarying trajectories) among different subjects may help to improve the understanding of personalized brain network development.

## Limitations and Future Work

In this study, we assume that all the non-overlapped meta-networks remain unchanged throughout the developmental process. However, it would be more realistic to take into account the temporal impact on the meta-networks. In other words, the meta-networks may evolve through different developmental stages (e.g., childhood, adolescence, and adulthood). Besides, we will compare the non-overlapped meta-networks with the overlapped ones and discuss the influence of the age group number in our future investigation. The current method can only deal with full developmental networks without any missing data. In fact, it would be more practical to extend the current method to handle incomplete within-subject longitudinal networks, because it is often the case that subjects only visit at a few time points. Additionally, we anticipate that a voxel-wise calculation could achieve a better result in terms of the meta-networks and covarying trajectories in a fine granularity. However, there will be a huge increase in computational load as there are millions of vertices. In the future, we will develop a more efficient way to deal with this problem. Moreover, different cortical atlases and more different cortical features would provide a broader view for the consistency of decomposition results. Therefore, in our future work, we plan to improve the meta-network decomposition method from the above respects and apply it to different types of neurodevelopmental network analysis.

## Conclusions

Our study provides insight into the developmental patterns of brain structural network from early childhood through early adulthood. To this aim, we develop a novel meta-network decomposition method that can give a consistent and compact representation for developmental brain networks. We demonstrate that the development of brain structural network is a smooth process that integrates multiple spatially heterogeneous meta-networks, which are dynamically weighted with their covarying trajectories. The intrinsic meta-networks reveal the underlying connection patterns that contribute to the dynamic change of brain network organization. Their corresponding covarying trajectories quantify the development of each meta-network, thus providing a benchmark for the development of healthy brain networks.

## Ethics Statement

All procedures performed in studies involving human participants were in accordance with the ethical standards of the institutional and/or national research committee and with the 1964 Helsinki declaration and its later amendments or comparable ethical standards. This article does not contain any studies with animals performed by any of the authors. Informed consent was obtained from all individual participants included in the study.

## Author Contributions

XX contributed to developing and implementing the method, visualizing, and analyzing the results. PH contributed to designing and analyzing the experiments, summarizing and visualizing the results, and writing the manuscript. P-TY and HZ contributed to the critical revision of the manuscript and the discussion of the work. JN contributed to preparing the data and revising the manuscript. DS contributed to directing the whole project.

### Conflict of Interest Statement

The authors declare that the research was conducted in the absence of any commercial or financial relationships that could be construed as a potential conflict of interest.

## Footnotes

^1^https://pediatricmri.nih.gov/nihpd/info/index.html
