# Supplementary material for: Meta-Network Analysis of Structural Correlation Networks Provides Insights Into Brain Network Development
Source: Front Hum Neurosci. 2019 Mar 26;13:93. doi: 10.3389/fnhum.2019.00093 (PMC6444117; doi:10.3389/fnhum.2019.00093)
Supplement: Supplementary file 1 [file Data_Sheet_1.pdf]

SUPPLEMENTARY INFORMATION:

Meta-network Analysis of Structural Correlation Networks Provides Insights into Brain Network Development

Xiaohua Xu[1], Ping He[1,*], Pew-Thian Yap[2], Han Zhang[2], Jingxin Nie[3], Dinggang Shen[2,4,*]

*** Correspondence:** Ping He (angeletx@icloud.com); Dinggang Shen (dgshen@med.unc.edu)

1 METHODS

1.1 Rich-club Structure

Rich-club is a highly connected hub core in a network that thought to enable efficient network communication. We evaluate the rich-club structures in meta-networks with the weighted rich-club coefficient Φ (Opsahl et al., 2008) and the normalized weighted rich-club coefficient Φ_{norm} (Colizza et al., 2006).

$$\Phi(k) = \frac{W_{>k}}{\sum_{l=1}^{E_{>k}} w_l^{\mathrm{ranked}}} \tag{1}$$

and

$$\Phi_{\mathrm{norm}}(k) = \frac{\Phi(k)}{\Phi_{\mathrm{rand}}(k)} \tag{2}$$

In eq. (1), $W_{>k}$ is the collective weight among the nodes with degrees larger than k in the network, $E_{>k}$ is the number of connections among the nodes with degrees larger than k, w_l^{ranked} is the weight of the lth connection in the ranked connections in descending order. In eq. (2), Φ_{rand} is the weighted rich-club coefficient averaged over a number of random networks with the same size and degree distribution as the evaluated meta-network. In this study, we generate 1000 random networks to estimate Φ_{rand} as a null model. If Φ_{norm} is consistently larger than the base line 1.0, then there is an evidence of rich club organization. Next, we perform a t-test (one-tailed) to examine whether Φ significantly exceeds Φ_{rand} for the range of k expressing rich-club organization (Van Den Heuvel and Sporns, 2011). If the p value of the t-test is less than a sufficiently small number (e.g., $p < 0.001$), then the rich-club structure is statistically significant in the evaluated meta-networks.

1.2 Covarying Trajectory and Degree Ratio

In this subsection, we will demonstrate the relationship between the covarying trajectories of the meta-networks and the degree ratios of brain regions in the developmental networks.

Let $\mathbf{X}^t \in \mathbb{R}^{78\times 78}$ represent the developmental brain network at the tth time point (e.g., at age of 3 years), $\mathbf{U}_i$ represent the ith meta-network, v_i^t represent the weight of the ith meta-network ($\mathbf{U}_i$) at the tth time point, $\mathbf{E}^t$ is the noise network at the tth time point. Based on our non-negative matrix factorization of the developmental networks, we have

$$\mathbf{X}^t = \sum_{i=1}^{r} v_i^t \mathbf{U}_i + \mathbf{E}^t \quad (3)$$

By multiplying the two sides of eq. (3) with an all-one vector $\mathbf{1} \in \mathbb{R}^{78}$, we have

$$\mathbf{X}^t \mathbf{1} = \sum_{i=1}^{r} v_i^t \mathbf{U}_i \mathbf{1} + \mathbf{E}^t \mathbf{1} \quad (4)$$

Note that $\boldsymbol{d}^t \triangleq \mathbf{X}^t\mathbf{1}$ is the degree vector of $\mathbf{X}^t$ that records the degrees of all the ROIs in $\mathbf{X}^t$, $\boldsymbol{d}_i \triangleq \mathbf{U}_i\mathbf{1}$ is the degree vector of $\mathbf{U}_i$ that records the degrees of all the ROIs in $\mathbf{U}_i$, and similarly $\mathbf{E}^t\mathbf{1}$ records the degree vector of $\mathbf{E}^t$. Next, we divide the two sides of eq. (4) by the total degree of $\mathbf{X}^t$, i.e., $\mathbf{1}^T\mathbf{X}^t\mathbf{1}$, leading to

$$\frac{\mathbf{X}^t\mathbf{1}}{\mathbf{1}^T\mathbf{X}^t\mathbf{1}} = \sum_{i=1}^{r} v_i^t \frac{\mathbf{U}_i\mathbf{1}}{\mathbf{1}^T\mathbf{X}^t\mathbf{1}} = \sum_{i=1}^{r} v_i^t \cdot \frac{\mathbf{1}^T\mathbf{U}_i\mathbf{1}}{\mathbf{1}^T\mathbf{X}^t\mathbf{1}} \cdot \frac{\mathbf{U}_i\mathbf{1}}{\mathbf{1}^T\mathbf{U}_i\mathbf{1}} + \frac{\mathbf{E}^t\mathbf{1}}{\mathbf{1}^T\mathbf{U}_i\mathbf{1}} \quad (5)$$

Note that $\boldsymbol{r}^t \triangleq \frac{\mathbf{X}^t\mathbf{1}}{\mathbf{1}^T\mathbf{X}^t\mathbf{1}}$ is the degree ratio vector of $\mathbf{X}^t$ that records the degree ratios of all the ROIs in $\mathbf{X}^t$, $\boldsymbol{r}_i \triangleq \frac{\mathbf{U}_i\mathbf{1}}{\mathbf{1}^T\mathbf{U}_i\mathbf{1}}$ is the degree ratio vector of $\mathbf{U}_i$, $\boldsymbol{r}_i$ is fixed through development, and $p_i^t \triangleq \frac{\mathbf{1}^T\mathbf{U}_i\mathbf{1}}{\mathbf{1}^T\mathbf{X}^t\mathbf{1}}$ is the ratio of the total degree of $\mathbf{U}_i$ to the total degree of $\mathbf{X}^t$. Since it has been demonstrated that the noise networks only take a very small proportion of the developmental networks (Figure 3(B)), we can discard the last term of eq. (5). Let $\hat{v}_i^t \triangleq v_i^t p_i^t$, eq. (5) can be rewritten in a more concise form,

$$\boldsymbol{r}^t = \sum_{i=1}^{r} v_i^t \cdot p_i^t \cdot \boldsymbol{r}_i = \sum_{i=1}^{r} \hat{v}_i^t \boldsymbol{r}_i \quad (6)$$

where $\hat{v}_i^t$ is the element of the weighted covarying trajectory at the tth time point. From eq. (6), we can see that the degree ratios in the developmental networks ($\boldsymbol{r}^t$) are linearly proportional to both the degree ratios in the meta-networks ($\boldsymbol{r}_i$) and the weighted covarying trajectories of the meta-networks ($\hat{v}_i^t$). If the kth region has much higher degree ratio in the ith meta-network than in the other meta-networks (i.e., $\forall j \neq i$, $\boldsymbol{r}_i^t(k) \gg \boldsymbol{r}_j^t(k) \to 0$), then $\boldsymbol{r}^t(k) \approx \hat{v}_i^t \boldsymbol{r}_i(k)$. Since $\boldsymbol{r}_i(k)$ is fixed through development, $\boldsymbol{r}^t(k) \propto \hat{v}_i^t$. Moreover, if the total degree of $\mathbf{X}^t$ keeps unchanged during development ($p_i^1 = p_i^2 = \cdots$), then $\boldsymbol{r}^t(k) \propto v_i^t$.

2 Results

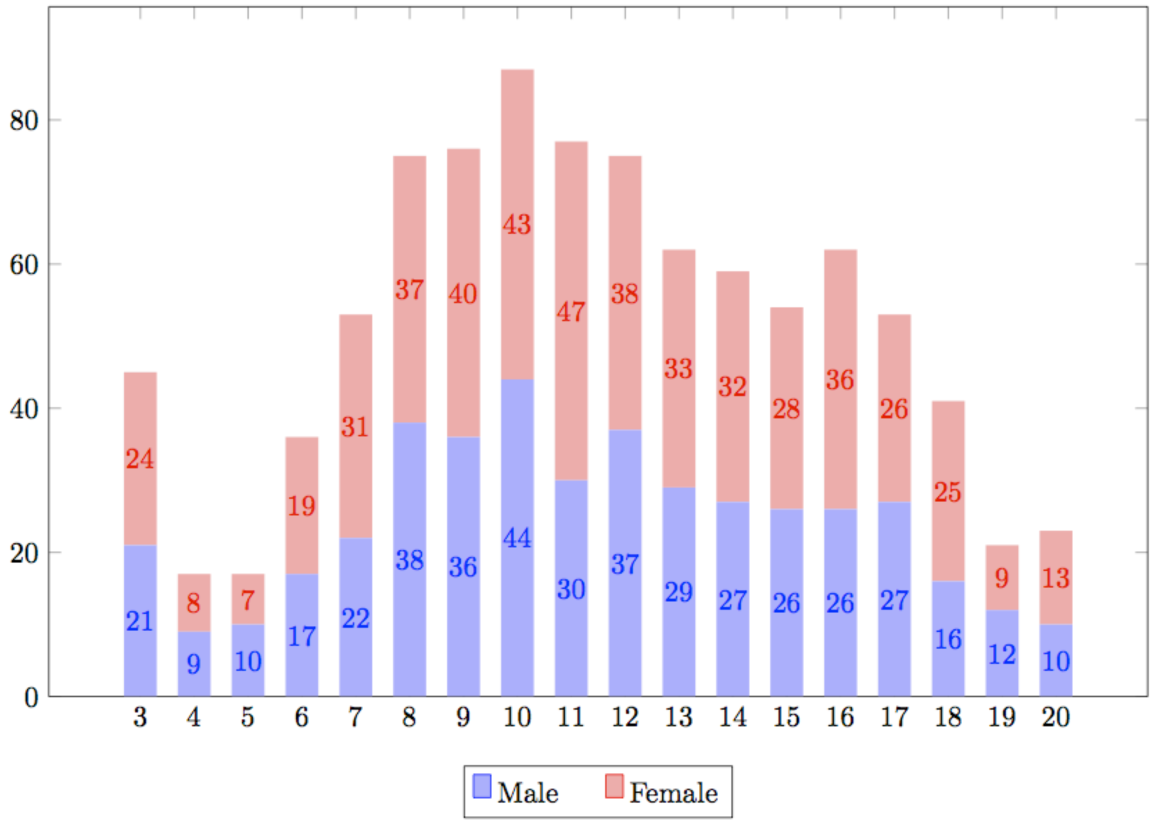

Figure S1. Session numbers of males and females at each age.

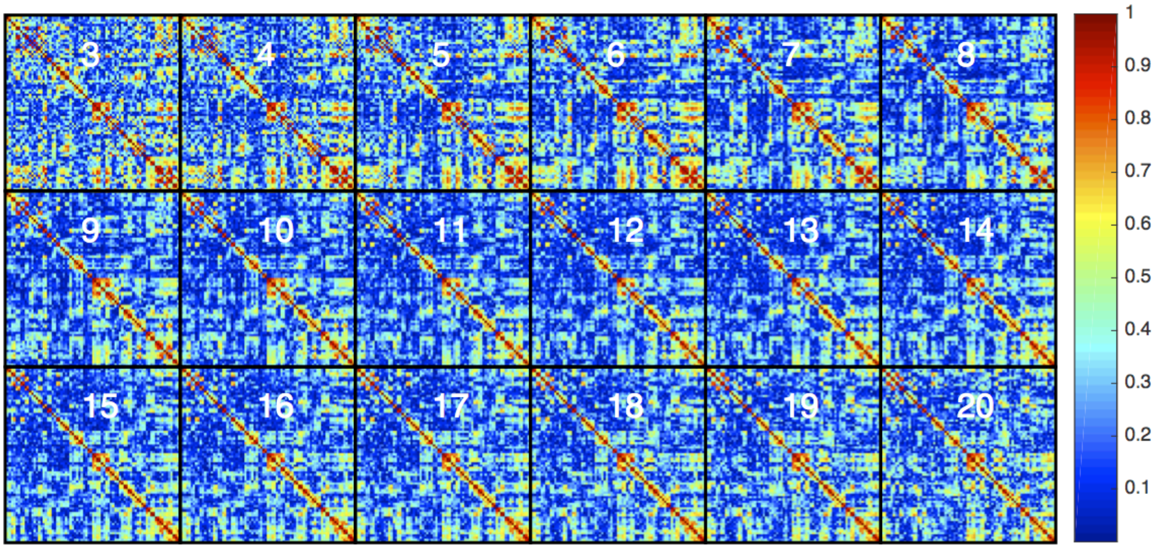

Figure S2. Developmental cortical thickness correlation networks from age 3 to 20.

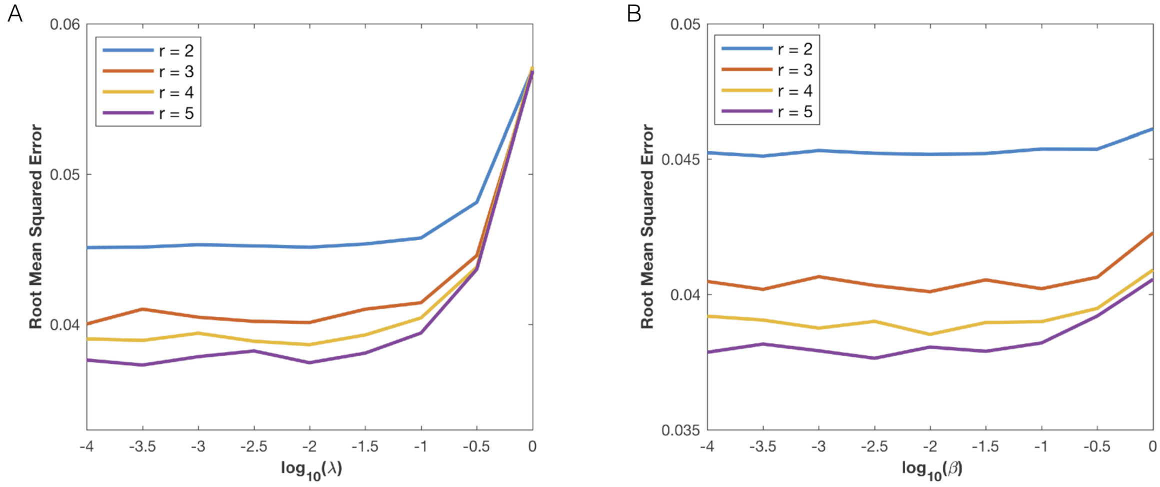

Figure S3: Meta-network decomposition is robust to a wide range of regularization parameters ($\beta \leq 0.1$ and $\lambda \leq 0.01$). (A) Influence of the parameter λ on the reconstruction error (root mean squared error) with fixed $\beta = 0.1$ and different meta-network numbers (r=2-5). (B) Influence of the parameter β on the reconstruction error (root mean squared error) with fixed $\lambda = 0.01$ and different meta-network numbers (r=2-5).

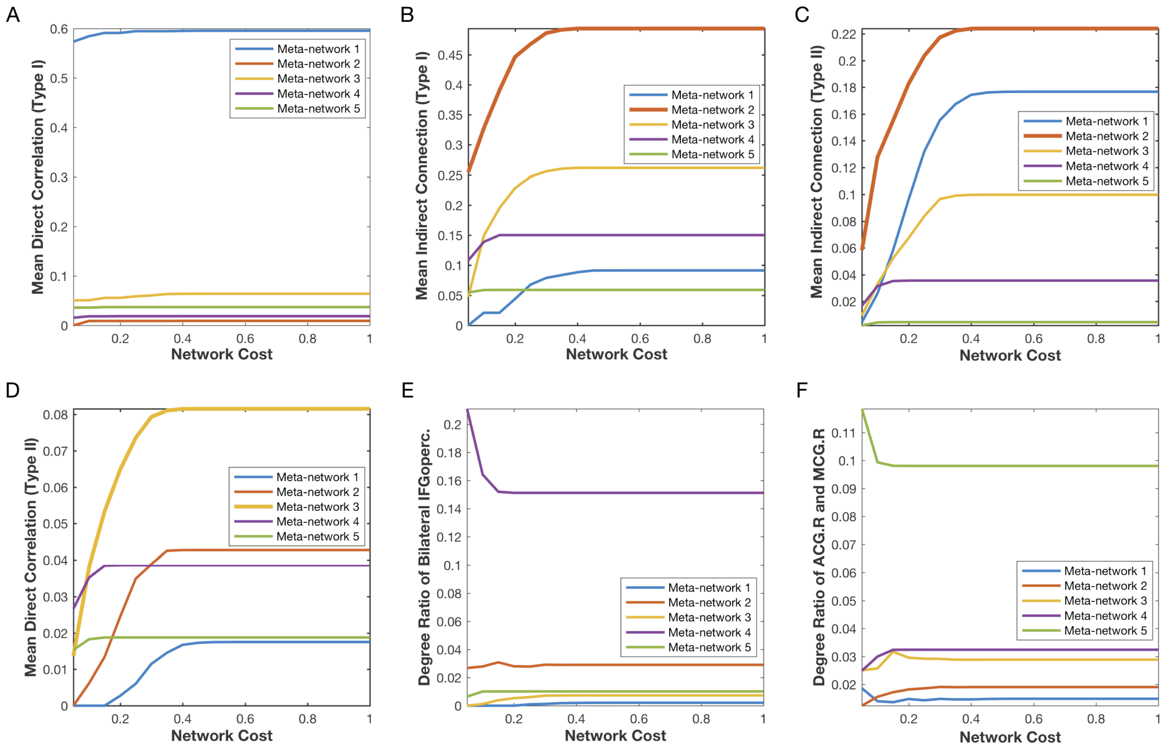

Figure S4: Quantitative validation of the connection patterns in the five meta-networks. (A) Meta-network 1 has the strongest mean direct correlation strength (type I) between the homotopic regions in bilateral hemispheres among the five meta-networks. The network cost is defined as the ratio of the number of reserved edges in a network to the maximum possible number of pair-wise correlations (78×78). (B) Meta-network 2 has the strongest mean indirect correlation strength (type I) between the homologous parietal regions through prefrontal cortices among the five meta-networks. (C) Meta-network 2 has the strongest mean indirect correlation strength (type II) between the prefrontal and occipital regions through frontal/temporal cortices among the five meta-networks. (D) Meta-network 3 has the strongest mean direct correlation strength (type II) between the prefrontal and occipital regions among the five meta-networks. (E) Meta-network 4 has the highest degree ratios of the bilateral IFGoperc., which are closely related with language processing, among the five meta-networks. (F) Meta-network 5 has the highest degree ratios of ACG.R and MCG.R, which are closely related with emotion control, among the five meta-networks.

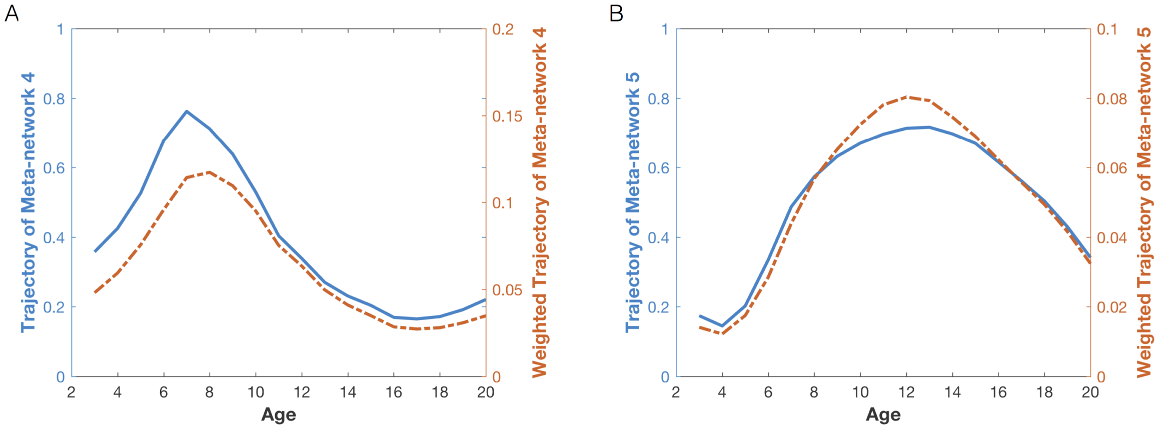

Figure S5: The covarying trajectories and the weighted covarying trajectories of Meta-networks 4-5. (A) The covarying trajectory and the weighted covarying trajectory of Meta-network 4 have very similar trend over development. (B) The covarying trajectory and the weighted covarying trajectory of Meta-network 5 have very similar trend over development.

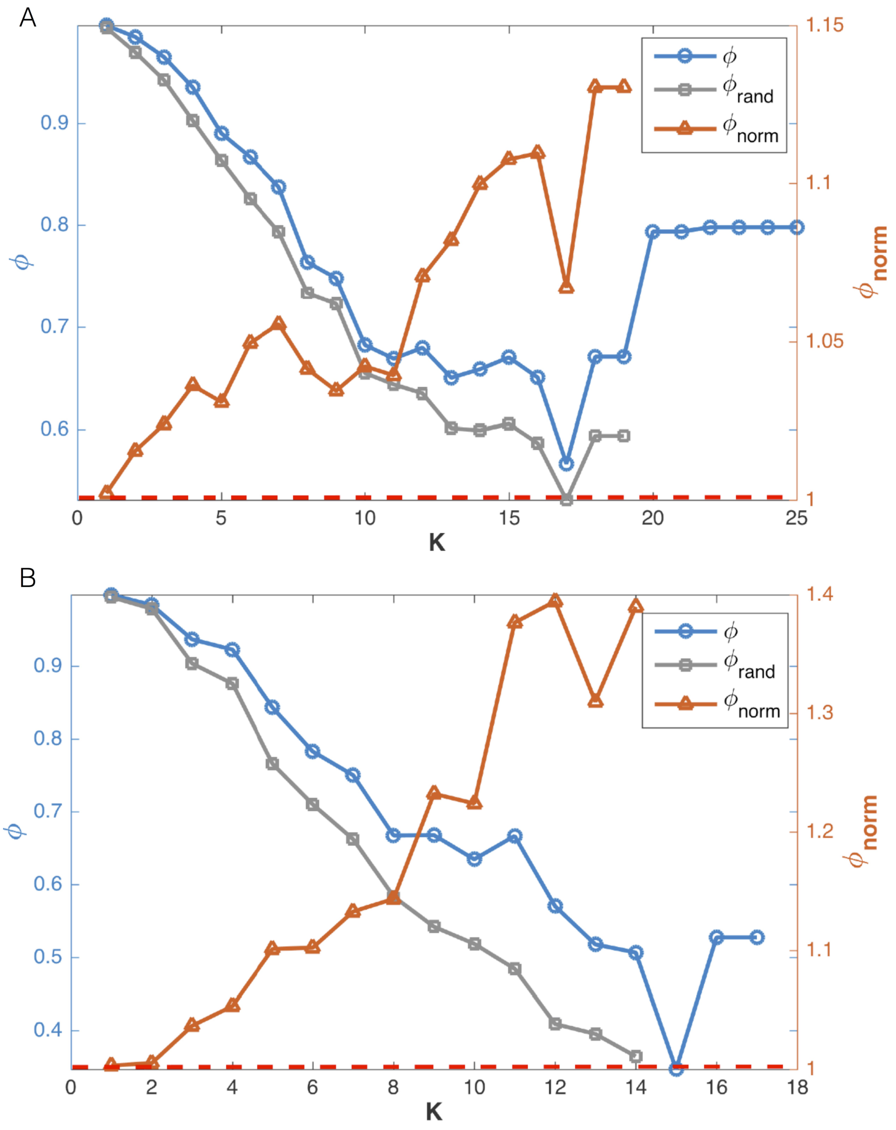

Figure S6: (A) Validation of rich-club structure in the fourth meta-network. Φ is the weighted rich-club coefficient of the fourth meta-network, Φ_{rand} (gray curve) is the weighted rich-club coefficient averaged over 1000 random networks with the same size and weight distribution as the fourth meta-network. The normalized rich-club coefficient Φ_{norm} (red curve) is consistently above the baseline 1.0 (red dashed line) since $k = 2$. The statistical test of $\Phi(k) > \Phi_{\mathrm{rand}}(k)$ for k = 2~19 (p < 0.001, one tailed) demonstrates the significance of rich club structure in the fourth meta-network. (B) Validation of rich-club structure in the fifth meta-network. Φ is the weighted rich-club coefficient of the fifth meta-network, Φ_{rand} (gray curve) is the weighted rich-club coefficient averaged over 1000 random networks with the same size and weight distribution as the fifth meta-network. The normalized rich-club coefficient Φ_{norm} (red curve) is greater than the baseline 1.0 since k = 3. The statistical test of $\Phi(k) > \Phi_{\mathrm{rand}}(k)$ for k = 3~14 (p < 0.001, one tailed) demonstrates the significance of rich club structure in the fifth meta-network.

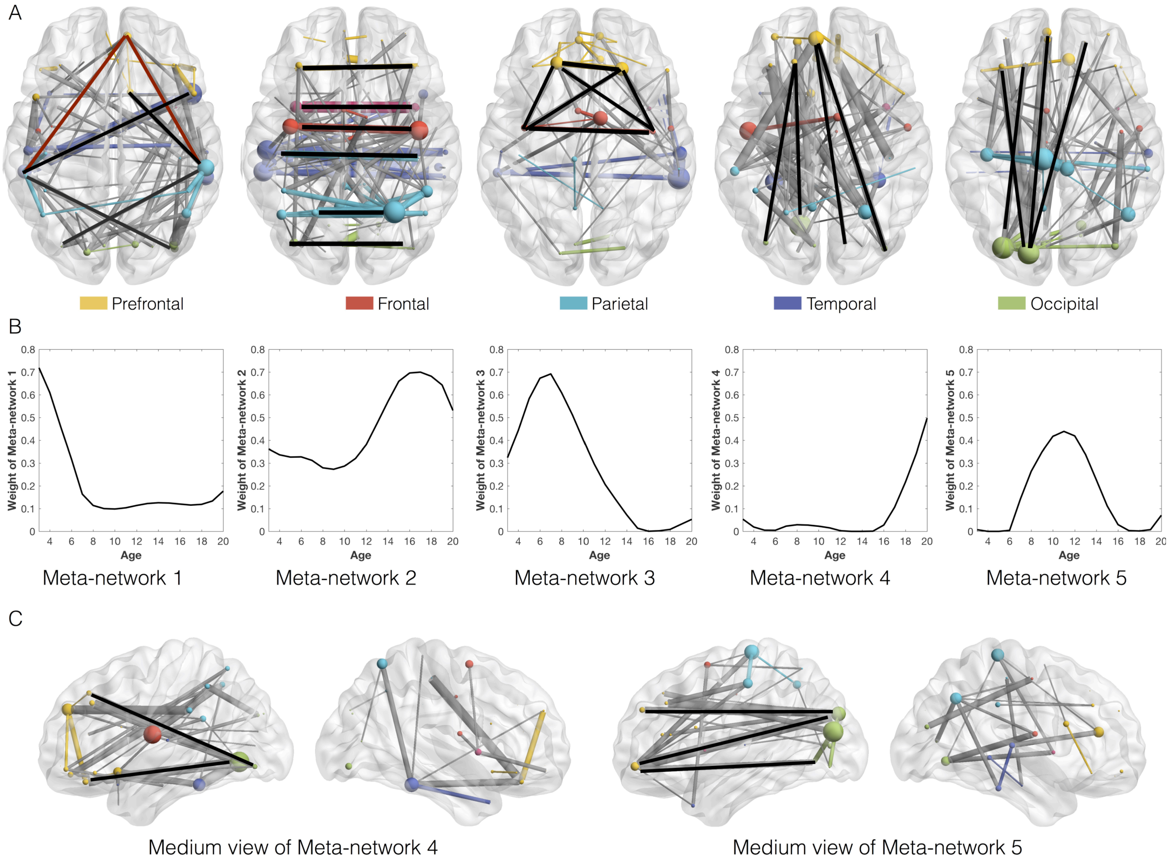

Figure S7: (A) Axial view of the five meta-networks decomposed from the developmental cortical-curvature correlation networks of the same subjects at 3-20 years of age. The size of each node (i.e., ROI) is proportional to its degree in the meta-network. The width of the edge is proportional to its correlation strength. The featured connections in each meta-network are highlighted in red/black lines. Specifically, the red lines in the first meta-network illustrate the indirect connections between the homotopic regions in two hemispheres, while the black lines illustrate the indirect connections between the prefrontal and occipital regions. In contrast, the lines in the second meta-network illustrate the direct connections between homotopic regions in two hemispheres. The third meta-network is featured with a local structure including both direct and indirect connections between homotopic regions in two hemispheres. The fourth and fifth meta-networks are highlighted with different types of direct connections between the prefrontal and occipital regions. (B) The covarying trajectories of the five cortical-curvature meta-networks move smoothly with the growth of age. The weight of the first meta-network, which includes two types of indirect connections, declines with the growth of age. On the contrary, the weight of the second and fourth meta-networks, which respectively include two types of direct connections, increases with the growth of age. (C) The medium view of meta-networks 4-5 presents a clearer illustration of the direct connections between prefrontal and occipital regions within the same hemispheres.

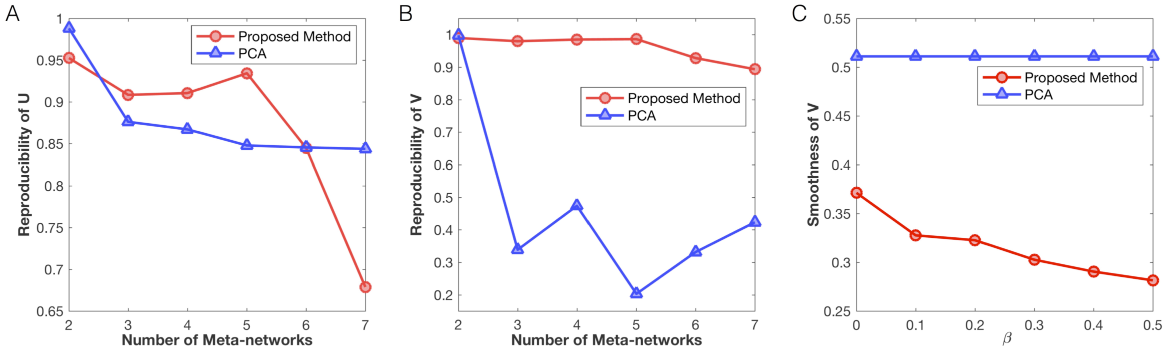

Figure S8. Comparison of the reproducibility and smoothness performance between the proposed method and PCA. (A) The reproducibility of the meta-networks (**U**) produced by our proposed method is higher than that of PCA when the meta-network number is within the range of [3,5]. (B) The reproducibility of covarying trajectories (**V**) produced by the proposed method is consistently higher than that of PCA. (C) The covarying trajectories of the proposed method are increasingly smoother than those of PCA as the weight of the smoothness constraint (β) grows larger. The smoothness of the covarying trajectories is quantified by computing the norm of the second order difference of **V**. The smaller value indicates the smoother covarying trajectories. Note that even when β =0, the covarying trajectories of the proposed method are still smoother than those of PCA.

References

Colizza, V., Flammini, A., Serrano, M., and Vespignani, A. (2006). Detecting rich-club ordering in complex networks. *Nature Physics* 2**,** 110 - 115.

Opsahl, T., Colizza, V., Panzarasa, P., and Ramasco, J.J. (2008). Prominence and control: the weighted rich-club effect. *Physical review letters* 101(16)**,** 168702.

Van Den Heuvel, M.P., and Sporns, O. (2011). Rich-club organization of the human connectome. *Journal of Neuroscience* 31(44)**,** 15775-15786.
